# Nematicidal Effect and Histological Modifications Induced by Hydrolysable Tannin Extract on the Third-Stage Infective Larvae of *Haemonchus contortus*

**DOI:** 10.3390/biology9120442

**Published:** 2020-12-04

**Authors:** Perla María del Carmen Acevedo-Ramírez, Claudia Hallal-Calleros, Iván Flores-Pérez, Fernando Alba-Hurtado, María Berenit Mendoza-Garfias, Rubén Barajas

**Affiliations:** 1 Faculty of Agronomy, Autonomous University of Sinaloa, Sinaloa 80090, Mexico; perlacevedoram@gmail.com; 2 Faculty of Agricultural Sciences, Autonomous University of Morelos State, Morelos 62209, Mexico; challalc@gmail.com (C.H.-C.); ivan.flores@uaem.mx (I.F.-P.); 3 Cuautitlan Faculty of Higher Studies, National autonomous University of Mexico, Estado de Mexico Cuautitlán Izcalli 54714, Mexico; fealba@hotmail.com; 4 Institute of Biology, National autonomous University of Mexico, Ciudad de México 04510, Mexico; berenit@ib.unam.mx

**Keywords:** anthelmintic, nematodes, functional food, parasites

## Abstract

**Simple Summary:**

*Haemonchus contortus* is the most frequent and most important nematode parasite in the ruminants production of tropical and subtropical regions. The conduction of research to find resources that allow effective control of this parasitic disease, preferably focusing on green production, is necessary. Hydrolysable tannins (HT) are an important group of compounds synthetized by plants, some of them exhibit anti-cancer, antioxidant, anti-inflammatory, anti-ulcerative, or antiparasitic properties. We evaluated the effect of hydrolysable tannin extract (HTE) on larvae of *H. contortus* (L_3_). L_3_ were exposed to different HTE concentrations and times to analyze the mortality, and microscopically we explored physical damage on L_3_ caused by HTE. After 72 h of incubation with 2 mg/mL of HTE, we found a half of death larvae, and by microscopy we observed larvae destruction. Our results suggest that hydrolysable tannin extracted from chestnut could be considered as an alternative for parasitic control as a food additive in cattle.

**Abstract:**

*Haemonchus contortus* is the most frequent and most important nematode parasite in the ruminants production of tropical and subtropical regions. There are strains resistant to all families of available anthelmintics. Consequently, the conduction of research to find other resources that allow effective control of this parasitic disease, preferably focusing on green production, is necessary. The aim of this study was to evaluate the effect of hydrolysable tannin extract (HTE) on larvae 3 (L_3_) of *H. contortus* in vitro. L_3_ were exposed to different HTE concentrations and times. In addition, both light and scanning electron microscopy were used to explore physical damage on L_3_ subjected to HTE activity. After 72 h of incubation, the mean lethal concentration of HTE was 2 mg/mL; this concentration has been previously referred to as safe for consumption in cattle. Scanning electron microscopy revealed *H. contortus* L_3_ destruction, damage was evident by separation of the sheath mainly in the cervical and caudal regions of the larva and by rupture of the cuticle with larval evisceration. Our results suggest that hydrolysable tannin extract from chestnut could be considered as a green alternative for parasitic control in ruminants.

## 1. Introduction

Gastrointestinal parasites cause decreased production and affect animal welfare [1]; among them, *Haemonchus contortus* (*H. contortus*) is the most frequent nematode parasite in ruminants in tropical and subtropical regions. This parasite affects animal health and production, deteriorating the quality of life and causing considerable economic losses. The direct economic losses are due to the decrease in the production of meat, milk and the death of the animals, and the indirect losses are attributable to the investment in control (anthelmintics, labor, equipment), reduction in the quality of the carcasses and predisposition to other diseases [2,3]. A meta-analysis indicates that, in gastrointestinal nematode-infected animals, the production in terms of weight gain, wool and milk is, respectively, 85, 90 and 78% of the production of parasite-free animals [4].

Natural polyphenols such as tannins have been proposed as alternatives for nematode control [5,6]. Despite the relative abundance of information about the use of condensed tannins [7,8], the available information relative to hydrolysable tannins is scarce. Engström et al. [9] performed an in vitro study using 33 hydrolysable tannins substances extracted using an 80% acetone aqueous solution at 4 °C from eleven plant species grown naturally in the southwest of Finland. As a conclusion, they affirmed that a relationship between the tannin structure and its anthelmintic activity exists, and ranked the ability of those compounds to inhibit *Haemonchus contortus* eggs’ hatchability and survival of L_1_ and L_2_ larvae. In that experiment [9], the authors attributed high anthelmintic activity to substances such as pentagallylglucose (82%) and tellmagrondin II (64%); at the same time, they ascribed very low values of relative anthelmintic activity to compounds such as castalagin (12%) and vescalgin (7%). However, those findings are not completely in concordance with those perceived by other researchers; Corona-Palazuelos et al. [10] fed a commercially available hydrolysable tannin extract obtained from chestnut (*Castanea sativa* Mill) to feedlot calves naturally infected with several parasites and observed that eggs excretion of *Haemonchus* sp. diminished by 61%. In addition, Acevedo-Ramírez et al. [11], using the same commercial hydrolysable tannin extract, observed a strong impact on in vitro mortality of *H. contortus* adults associated with severe morphological changes. Paradoxically, castalagin and vescalgin represent the larger concentration of hydrolysable tannin substances present in commercial chestnut extract [12,13]. Chestnut is the first worldwide-available commercial source of hydrolysable tannin extract [12,14] and the third as a tannins source after condensed tannins-rich plants of quebracho trees (*Schinopsis lorentzii*) and mimosa trees (*Acacia mearnsii* De Willd) [15,16]. Therefore, it becomes of interest to test if tannins extract from chestnut is effective against the infectious-stage larvae 3 of *H. contortus*, similar to when it is effective against the adult parasite. Otherwise, the assumption of Engström et al. [8] that tannins extract predominantly with a content of substances such as castalagin and vescalgin has no anthelmintic activity against larvae of *H. contortus* would be corroborated. Therefore, the aim of the current research was to evaluate the in vitro effect of a hydrolysable tannin (HT) extract from chestnut on the stage-three larvae of *H. contortus*.

## 2. Materials and Methods

### 2.1. Ethic Statements

This work does not involve the direct use of animals by any of the authors.

### 2.2. Parasites

A strain of larvae 3 of *H. contortus* was supplied by the Department of Parasitology in the Faculty of Higher Studies Cuautitlán, UNAM. Larvae were isolated and characterized as previously described [17]. Briefly, feces were collected from a lamb inoculated with larvae of *H. contortus*; L_3_ were obtained from feces culture and washed with distilled water to recover living and active specimens. The isolate used for lamb inoculation was characterized and used in previous studies [16], and the high virulence, fertility and morphology of the strain were periodically verified.

### 2.3. Hydrolysable Tannin Extract (HTE)

The hydrolysable tannins were obtained from a commercially available preparation containing 75% of hydrolysable tannins extracted from chestnut tree (Silvafeed Nutri P^®^, SilvaTeam, San Michele Mondovi, Italy). A stock solution was prepared at 100 gr/Lt in tap water and filtered with Whatman^®^ (Merck Life Sciences UK. Dorset, England) paper. Different concentrations of HTE (0, 1, 2, 4, 8, 50 and 100 mg/mL) were prepared from the stock solution by diluting it in tap water [10].

The HTE doses tested were calculated using prediction formulas proposed by NASEM [18], based on results described by Corona-Palazuelos et al. [9]. They used bull calves of 231.6 kg mean bodyweight fed a diet containing 1.358 Mcal NEm/kg DM, including 1.5% of HTE.

From these data, daily dry matter intake (DMI) was calculated as 5.59 kg in accordance with a prediction formula: NEm Intake = SBW^0.75^ × (0.2435 × N − 0.0466 × NEm^2^ − 0.1128), where NEm Intake is the total daily NEm intake in Mcal, SBW^0.75^ is SBW = BW × 0.96 and NEm is the NEm content of the diet in Mcal/kg DM. In that research, the daily HTE intake was 44.72 (experiment 1) and 83.85 g by bull calves (experiment 2). Daily drinking water intake was calculated as 22.4 L using the formula DWI, L/day = −6.0716 + (0.70866 × Maximum Temperature °C) + (2.432 × DMI kg/day) − (3.87 × Pluvial precipitation mm) − (4.437 × Salt in diet as %). Therefore, 44.72 g/22.4 L = 2.0 g/L, equivalent to 2 mg/mL (experiment 1); and 83.85 g/22.4 L = 3.74 g/L (3.74 mg/mL), which was rounded to a 4 mg/mL dose (experiment 2).

### 2.4. Bioassays

Solutions containing different concentration (0, 1, 2, 4, 8, 50 and 100 mg of HTE/mL) were placed in 96-well plates in triplicate, containing approximately 200 ensheathed L_3_/well, kept at 37 °C and 5% CO_2_ for 24, 48, 72 and 96 h (3 wells by treatment). The whole procedure was performed in triplicate and repeated on three different days (nine wells by each concentration in total). After exposure to the treatment, aliquots of L_3_ were taken and observed on a slide under an optical microscope, and 100 larvae were counted per treatment. Larvae showing movement were considered alive; larvae that did not show perceptible movement even under the most intense light exposure of the microscope were considered dead. In vitro mortality of *H. contortus* L_3_ was expressed as percentage (percentage of mortality = (number of death cells/number of total cells) × 100). The movement of L_3_ from each well was videotaped directly from the culture plates using an inverted microscope (Videos S1–S3).

### 2.5. Scanning Electronic Microscopy (SEM)

For the preparation of specimens for SEM, L_3_ were exposed to different concentrations of HTE for distinct times, fixed using 4% formalin for 24 h and washed twice for 10 min with distilled water and twice with saline solution. Progressive dehydration with graded ethanol solutions for 1 h was performed, with three subsequent washes (1 h each) using 100% ethanol. A dried critical point process was performed utilizing extra-dry CO_2_. Dehydrated L_3_ were assembled on carbon adhesive aluminum slides and covered with a 20 mA gold layer for 2 min. Samples were observed with a Hitachi SU1510^®^ (Hitachi City, Japan) scanning electronic microscope at 10 to 15 kw [19].

### 2.6. Statistical Analyses

For the analysis of the percentage of mortality, an analysis of variance was performed with a completely randomized design with a factorial arrangement of 7 × 4 (seven doses and four incubation times). The time of incubation and separation of means were analyzed with the Tukey test, and the *alpha* level was 0.01 using version 9 of the Statistix program (Analytical Software, Tallahassee, FL, USA) The 50 and 90 lethal concentrations (LC_50_ and LC_90_) were obtained by interpolation of the mortality curves.

## 3. Results

### 3.1. HTE Induced Mortality on L_3_ Larvae of H. Contortus

**Figure 1:**
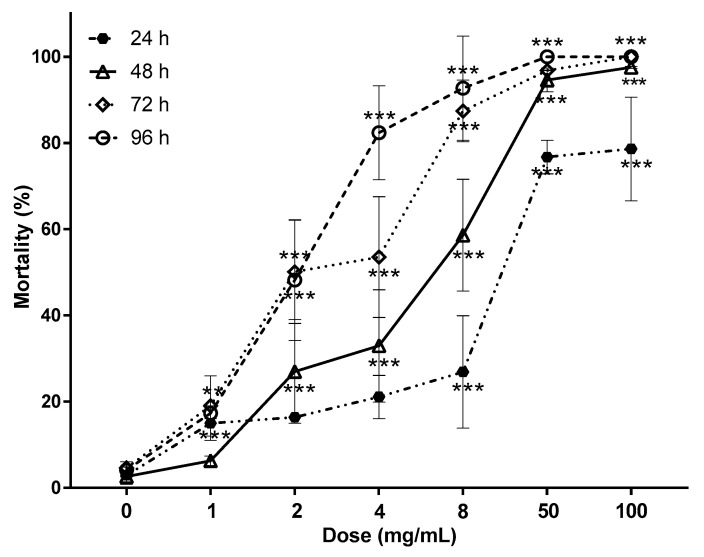
Concentration- and time-dependent response of larvae 3 (L_3_) to hydrolysable tannins. Percentage of *H. contortus* L_3_ mortality exposed to different concentrations of hydrolysable tannins for 24, 48, 72 and 96 h (mean ± SE, *** *p* ≤ 0.0001, ** *p* ≤ 0.001 one-way ANOVA, Tukey–Kramer post-test, comparison between concentrations).

The influence of hydrolysable tannin extract concentration and exposition time on mortality of stage-three larvae of *H. contortus* is shown in Figure 1. An interaction between HTE dose and exposition time was found (*p* < 0.0001), where mortality was increased over time with the HTE concentration increasing from 2 to 8 mg/mL, while mortality in the control group at 0.0 mg HTE/mL was similar from 24 until 96 h (*p* > 0.10). The mortality of *H. contortus* was concentration-dependent in a linear manner at any incubation time (*p* < 0.0001). After 24 h of incubation, concentrations of 50 and 100 mg/mL of HTE induced mortality higher than 75%. At 72 h, larvae subjected to 100 mg/mL of HTE reached 100% of mortality, and concentrations of 8 and 50 mg HTE/mL produced similar results (*p* > 0.10). After 96 h of larvae incubation with HTE, 4 mg/mL was enough to obtain similar results to those obtained with 100 mg/mL (*p* > 0.10) at 72 h.

### 3.2. Lethal Concentration of HTE on L_3_ of H. Contortus

**Figure 2:**
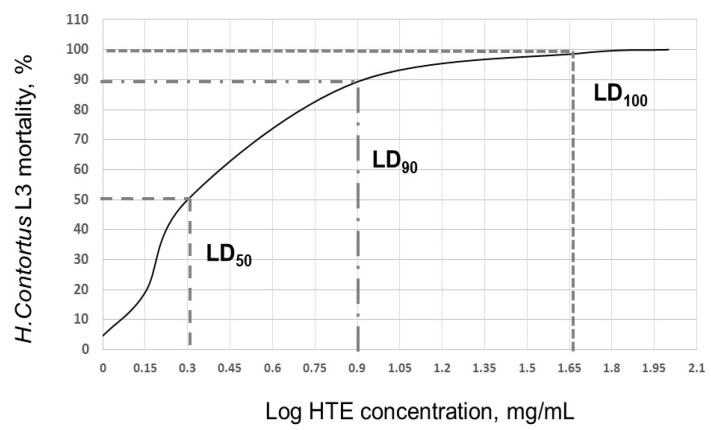
Lethal concentration (LC) of hydrolysable tannin extract (THE) on *H. contortus* L_3_. Semi-logarithmic curve for HTE LC_50_ and LC_90_ determination at 72 h post-treatment.

The lethal concentrations were dependent on the treatment time, finding an LC_50_ of 25 mg/mL at 24 h, 6 mg/mL at 48 h and 2 mg/mL at 72 and 96 h. The LC_90_ was reached with 40 mg/mL at 24 h, decreasing to 8 mg/mL at 72 h and to 5 mg/mL at 96 h of treatment. At 72 h of incubation, the mean lethal concentration (LC_50_) of HTE was 2 mg/mL (Antilog 0.3 = 1.995) and the LC_90_ was 8 mg/mL (Antilog 0.9 = 7.943) (Figure 2).

### 3.3. Damage of L_3_ Induced by HTE Observed by Optical Microscopy

**Figure 3:**
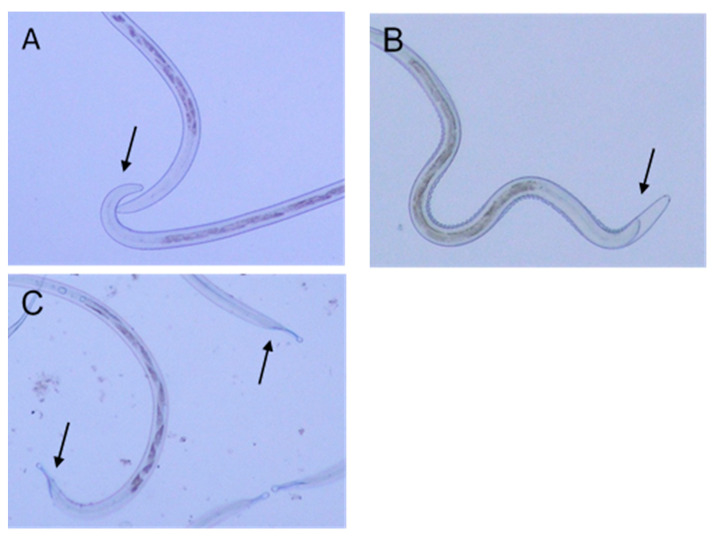
Damage induced by HTE on L_3_ observed through optical microscope. Microphotographs of L_3_ of *H. contortus* incubated for 96 h. (**A**) Control. (**B**) 1 mg/mL, separation of the sheath and retraction of the larval body (arrow). (**C**) 8 mg/mL, separation and folding of the sheath at the cephalic end and at the caudal end of the larva (arrow).

By observation under an optical microscope, we were able to record the damage induced by the HTE on L_3_. In the control group, we observed a morphology without alterations (Figure 3A), whereas in the treated groups, with a concentration of 1 mg/mL at 96 h, a separation of the sheath was observed mainly in the cephalic region (Figure 3B), and as the concentration was increased to 8 mg/mL, a dehydration that caused the collapse of the sheath of the cephalic and caudal regions was observed (Figure 3C).

### 3.4. Damage of L_3_ Induced by HTE Observed by Scanning Electron Microscopy

**Figure 4:**
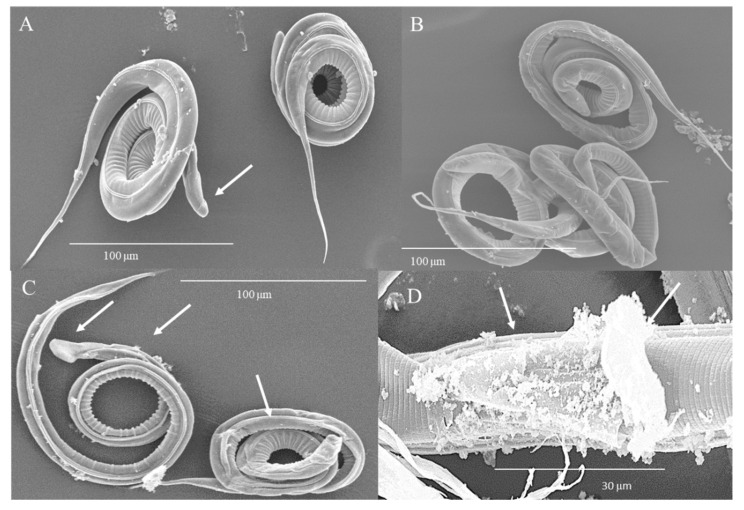
Microphotographs of L_3_ of *H. contortus* incubated with HTE for 24 h. (**A**) Control, cephalic end without alteration. Scale bar: 100 µm (**B**) 8 mg/mL, folding and retraction of the sheath and cuticle. Scale bar: 100 µm. (**C**) 50 mg/mL, loss of sheath and of cuticle integrity (arrows). Scale bar: 100 µm. (**D**) 100 mg/mL, extensive loss of sheath and cuticle integrity, flaking and vacuolization (arrows). Scale bar: 30 µm.

Figure 4 details the damage observed by SEM on the structure of the parasite after 24 h of HTE treatment. In control parasites (Figure 4A), the cephalic end is shown without alteration. Treatment with 8 mg/mL induced folding and retraction of the sheath and cuticle of the body of the larva. At 50 mg/mL, loss of sheath and cuticle integrity is observed, mainly at the cephalic end (arrows). At 100 mg/mL, extensive loss of sheath and cuticle integrity accompanied by flaking and vacuolization at the cranial end was induced (Figure 4B–D).

**Figure 5:**
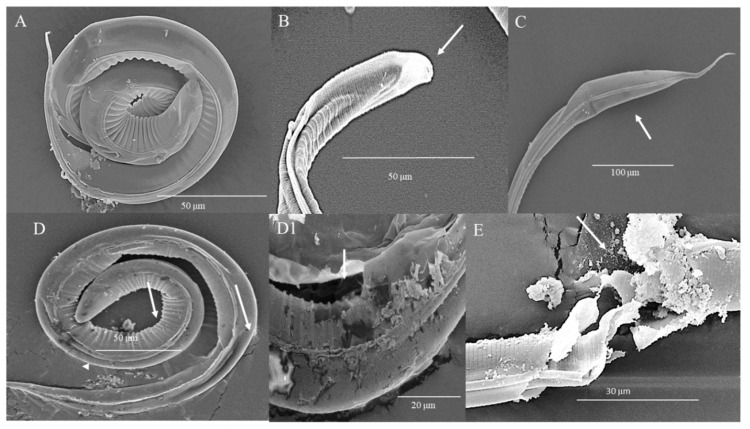
Microphotographs of L_3_ of *H. contortus* incubated with HTE for 48 h. (**A**) Control, intact sheath and cuticle. Scale bar: 50 µm. (**B**) 1 mg/mL, swelling of the cranial end. Scale bar: 50 µm. (**C**) 8 mg/mL, caudal end with laminar structure, increased size and cuticular detachment. Scale bar: 100 µm. (**D**,**D1**) 50 mg/mL, loss of the integrity of the cuticle in the form of furrows, cuticular erosion, flaking and vacuolization. Scale bar: 50 µm, 20 µm. (**E**) 100 mg/mL, cuticular and larval lysis. Scale bar: 30 µm.

After 48 h of incubation with HTE (Figure 5), parasites incubated with water remained with an intact sheet and cuticle (Figure 5A). Larvae incubated with 1 mg/mL of HTE increased in volume (probably by turgor) at the cranial end (Figure 5B). While the concentration was increasing, damage was observed in the caudal end with a laminar structure, increased size and cuticular detachment (Figure 5C); loss of the integrity of the cuticle was observed in the form of furrows, as well as cuticular erosion with a generalized distribution in the body of the larva, associated with flaking and vacuolization (Figure 5D,D1); and cuticular and larval lysis, associated with damage to intestinal cells and internal structures, with a generalized rupture of the body was caused with the higher concentration tested (Figure 5E).

**Figure 6:**
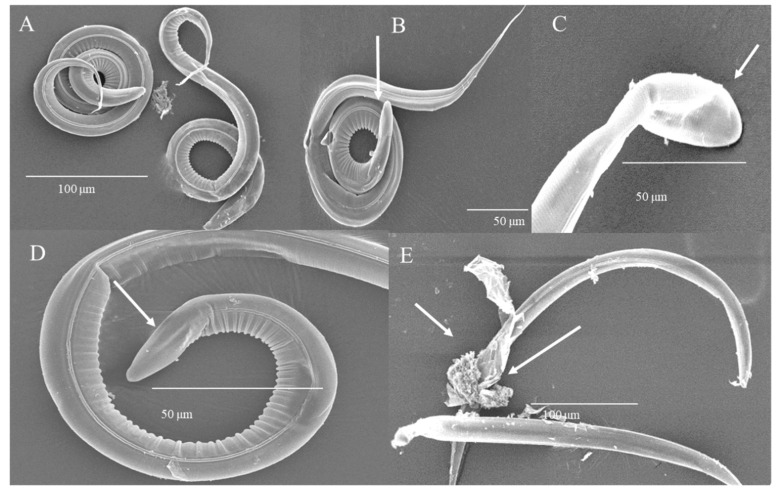
Microphotographs of L_3_ of *H. contortus* at 72 h. (**A**) Control, intact larvae. Scale bar: 100 µm. (**B**) 1 mg/mL, detachment of the sheath and cuticle at the cranial end of the larva as well as widespread destruction of cuticular integrity. Scale bar: 50 µm. (**C**) 4 mg/mL, cuticular detachment at the cephalic end associated with increased size. Scale bar: 50 µm. (**D**) 8 mg/mL, cuticular detachment at the cephalic end and generalized flaking. Scale bar: 50 µm. (**E**) 50 mg/mL, loss of integrity of the cuticle with protrusion of internal digestive structures and lysis of the larvae. Scale bar: 100 µm.

The morphology of L_3_ observed through SEM after 72 h of treatment (Figure 6) showed intact control larvae (Figure 6A), while in larvae treated with 1 mg/mL, detachment of the sheath and cuticle at the cranial end of the larvae as well as widespread destruction of cuticular integrity is observed (Figure 6B). At 4 mg/mL, cuticular detachment at the cephalic end associated with increased size is observed (Figure 6C). Treatment with 8 mg/mL induced cuticular detachment at the cranial end and generalized flaking (Figure 6D), while 50 mg/mL induced loss of integrity of the cuticle with protrusion of internal digestive structures and lysis of the larvae (Figure 6E).

**Figure 7:**
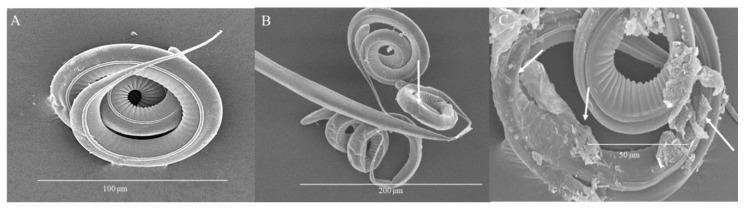
Microphotographs of L_3_ of *H. contortus* at 96 h. (**A**) Control, intact larvae. Scale bar: 100 µm. (**B**) 1 mg/mL, folding and cuticular lysis. Scale bar: 200 µm. (**C**) 4 mg/mL, flaking, vacuolization and protrusion of internal digestive structures and partial lysis. Scale bar: 50 µm.

The morphology of L_3_ observed through SEM after treatment with HTE for 96 h is observed in Figure 7. Control larvae were intact (Figure 7A), while larvae treated with 1 mg/mL showed folding and cuticular lysis to a greater extent associated with a focused tear. At 4 mg/mL, flaking, vacuolization and protrusion of internal digestive structures as well as partial lysis of the larvae were observed.

## 4. Discussion

Concentrations for an in vitro assay are very difficult to precisely extrapolate from in vivo conditions and vice versa. Although several models have been proposed, including pharmaceutical mathematical models capable of predicting in vivo efficacy, reliable predictions and extrapolation remain a challenge. In this work, the HTE concentrations tested were based on in vivo results described by Corona-Palazuelos et al. [10], where the authors fed bull calves with a diet containing 1.5% of HTE, and observed a reduction in the amount of nematodes. Based on those concentrations, we used a prediction formula proposed by NASEM [18], showing that the doses administered in vivo are equivalent to 2 (experiment 1) and 4 mg/mL (experiment 2).

Here, mortality of *H. contortus* L_3_ was observed as a consequence of exposition to chestnut HTE, even at low concentrations such as 2 mg/mL if given for enough time (72 h). The mortality increment is concentration- and time-dependent and is strong evidence of anthelmintic activity, as appeared from the substantial structural damage revealed by the electron microscopic study. The proposed mechanism for the activity of hydrolysable tannins is through binding to collagen proteins and proteins rich in proline and hydroxyproline on the cuticle of the larvae [9].

Several authors proposed the use of plants rich in tannins as a potential alternative for the decrease in the motility and migration of *H. contortus* larvae [20,21,22], and also due to its contribution to reduce the establishment of larvae in the host [23,24]. Athanasiadou et al. [7] observed that with concentrations from 0.81 to 3.32 mg/mL of condensed tannin extract (CTE) from quebracho tree, the viability of *H. contortus* larvae decreased after six days of exposure.

Engström et al. [9] obtained 33 compounds chemically characterized as hydrolysable tannins from twelve plants from Southwest Finland. They tested their activity against L_1_ and L_2_ of *H. contortus* larvae and against eggs and identified structural changes through SEM, mainly on the body surface (cuticle) and in the cephalic region of L_1_ and L_2_. We reported other apparent structural changes in L_3_, such as longitudinal and transverse folds and thicker crests in the cuticle. Engström et al. [9] observed aggregates located in the mouth capsule and in the anterior amphidial channels in L_1_ and L_2_. In the current study, we identified damage in L_3_ that caused a delamination of the cuticle and even its rupture with evisceration. As a conclusion of their experiments, Engström et al. [9] postulated a relationship between some structural aspects of hydrolysable tannins and their anthelmintic activity and proposed a ranking. At the bottom of the ranking, they included substances with very low activity against *H. contortus* including compounds such as castalagin with 12% and vescalgin with only 7% of theoretical anthelmintic activity. Precisely, castalagin and vescalgin represent the largest concentration of hydrolysable tannin substances present in commercial chestnut extracts [12,13], as used in the current experiment.

The observed structural damage in both experiments is in discordance with the low responses predicted by Engström et al.’s [9] data and the anthelmintic activity we found in the chestnut extract. These opposite results corroborate the concept that tannins extracted from different plants show differences in their properties, and that plant species, plant origin, phonologic state of the plant and method of extraction have an influence on tannins’ activity [13,15]. It is important to take into account that most of the experiments use tannins obtained from leaves, stems and buds of herbs and legumes with annual growth behavior, and that the final content of wood tannins is influenced by environmental conditions and by extracting procedures which vary from laboratory to laboratory [25]. Nevertheless, commercial tannin extracts are commonly obtained from trunks of trees around 30 years old, and branches are discarded [16]. In the trunks, after several years, natural variation in the tannins concentration due to climate decreases and the final wood tannins content tends to be homogeneous. The industrial process of extraction contributes to standardizing the type and amount of tannins found in the finally delivered tannin extract. The extract produced industrially on a large scale maintains constant quality and this provides an advantage for ruminant feed [15], thus the reason why we used a commercially available hydrolysable tannin extract from chestnut (Silvafeed Nutri P^®^) in the current experiment. Nutri-P contains 75% of tannin extract obtained from chestnut (Silvafeed 2011, Product data sheet Nutri-P, Queretaro, Mexico). These data are in agreement with commercial chestnut extract information described in the scientific literature. The normal tannin content of spray-dried chestnut extract is 75% when measured according to the filter method. Typical analytical values are: 75% of tannins, 17.6% of nontannin, 0.4% of insoluble and 7% of water content with a pH of 3.5. Approximately 89% of the tannin components are the hydrolysable tannins castalagin, vescalagin, castalin and vescalin [12]. A more detailed and quantitative analysis of the compounds of sweet chestnut commercial dry fractions was described by Campo et al. [13], who found vascalagin 45.2 mg/g, castalagin 39.7 mg/g, O-galloyl-castalagin isomer 32.0 mg/g, gallic acid 13.6 mg/g, digalloyl glucose 12.2 mg/g, trigalloyl glucose 12.1 mg/g, pedunculagin I 10.1 mg/g, roburin D 9.6 mg/g, tetragalloyl glucose 9.2 mg/g, castalin 8.8 mg/g, vescalin 8.7 mg/g, ellagic acid 7.8 mg/g, dehydrated tergallic-C-glucoside 6.0 mg/g, monogalloyl glucose II 5.0 mg/g and monogalloyl glucose I 4.7 mg/g

From our L_3_ mortality data, we calculated that the in vitro LC_50_ at 72 h of exposition was 2 mg/mL. After 96 h, mortality of *H. contortus* L_3_ with a dose of 4 mg/mL was close to the 100% (*p* > 0.10) that was observed with 50 and 100 mg/mL at the same exposure time.

Féboli et al. [26] reported an ovicidal effect using extracts of *Opuntia ficus*; they attributed the antiparasitic effects to the alkaloids, tannins, flavonoids and saponins in the fruits and cladodes. However, variations in secondary metabolites occur due to the age of the plant, environmental conditions and maturation stage of the fruits. In contrast, we tested a standardized wood tannin extract that allows a constant dosage, which has been tested in vivo with favorable results in the reduction in the feces egg count of *H. contortus* [10] and also on the adult stage of *H. contortus* [11].

Although tannin concentrations used in our in vitro study were based on a calculation model of NASEM, an effective concentration can still not be extrapolated to an in vivo application since we did not test conditions in which dietary proteins would interact with tannins in a ruminant digestive tract, such as different inorganic ion conditions or different pH conditions that prevail in different phases of the gastrointestinal tract. It is well known that molecules can form complexes with other small molecules or with macromolecules such as proteins; once complexation occurs, the physical and chemical properties of the complexing species are altered and complexation can alter the pharmacologic activity. Some altered properties include solubility, stability, partitioning, energy absorption and emission and conductance of the molecules [27]. Complexation, therefore, can lead to beneficial properties such as enhanced aqueous solubility and stability; in some cases, it can lead to poor solubility or decreased absorption. Further, complexation with certain hydrophilic compounds can enhance excretion. The most familiar tannin–protein interactions result in precipitation of the complex, but soluble complexes also form under certain conditions. Both soluble and insoluble complexes are stabilized by reversible, noncovalent bonds between the tannin and protein [28]. Tannin–protein interactions are influenced by characteristics of the protein (including size, amino acid composition, pI and extent of post-translational modification), characteristics of the tannin (size, structure, heterogeneity of the preparation) and conditions of the reaction (pH, temperature, solvent composition, time); therefore, results of an in vitro study must be taken cautiously.

In our work, the mobility of the larvae was qualitatively observed; it was affected at the lowest concentrations of HTE and at the shortest incubation times. When incubated in the control solution, the larvae maintained the characteristic undulating movements, not affected throughout the 96 h of observation (Video S1). The larvae incubated with 2 mg/mL of HTE for 24 h decreased their movement (Video S2), and, at higher concentrations, we observed total paralysis and immobilization of the larvae (Video S3).

Our work confirms the nematicidal activity of standardized chestnut wood tannins and helps to elucidate the mechanism of action of these compounds, showing that they have toxic action on the L_3_ of the parasite. It is important to highlight that the use of these tannin extracts as anthelmintics is promising as food additives and as an adjuvant to achieve the reduction in the burden of worms. However, to propose a future anthelmintic drug, it is necessary to perform more studies, including the search for a major component with antiparasitic activity that could be isolated or synthesized for pharmacological purposes. Isolation of the main components will allow the study of accurate doses administration, reducing or eliminating the toxic effects due to impurities in the plant products; moreover, knowledge of the chemical structure of the pure components will enable the laboratory synthesis of many structurally related compounds and the development of valuable drugs.

## 5. Conclusions

The in vitro study of hydrolysable tannin extract showed that it causes a decrement in motility with the subsequent death of L_3_. The effect was concentration- and time-dependent, and the observed structural damage suggests that the hydrolysable tannins used in this study can be an alternative for parasitic control as a food additive in cattle.

## Supplementary Materials

Videos S1–S3: http://doi.org/10.5281/zenodo.4292708. Video S1: Control larvae, Video S2: Larvae treated with 2 mg/ml of HTE for 24 h, Video S3: Larvae treated with 100 mg/ml of HTE for 72 h.
